# Endocytic Uptake of Solid Lipid Nanoparticles by the Nasal Mucosa

**DOI:** 10.3390/pharmaceutics13050761

**Published:** 2021-05-20

**Authors:** Ammar S. Al Khafaji, Maureen D. Donovan

**Affiliations:** Department of Pharmaceutical Science and Experimental Therapeutics, College of Pharmacy, University of Iowa, 115 S. Grand Avenue, Pharmacy Building, Iowa City, IA 52242, USA; ammar-alkhafaji@uiowa.edu

**Keywords:** solid lipid nanoparticles, endocytosis, intranasal drug delivery

## Abstract

Nanoparticles may provide unique therapeutic opportunities when administered via the nasal cavity, yet the primary uptake and transfer pathways for these particles within the nasal mucosa are not well understood. The endocytic pathways involved in the uptake of fluorescently labeled, (Nile Red) solid lipid nanoparticles (SLNs) of different sizes (~30, 60, and 150 nm) were studied using excised bovine olfactory and nasal respiratory tissues. Endocytic activity contributing to nanoparticle uptake was investigated using a variety of pharmacological inhibitors, but none of the inhibitors were able to completely eliminate the uptake of the SLNs. The continued uptake of nanoparticles following exposure to individual inhibitors suggests that a number of endocytic pathways work in combination to transfer nanoparticles into the nasal mucosa. Following exposure to the general metabolic inhibitors, 2,4-DNP and sodium azide, additional, non-energy-dependent pathways for nanoparticle uptake were also observed. While the smallest nanoparticles (30 nm) were the most resistant to the effects of pharmacologic inhibitors, the largest (150 nm) were still able to transfer significant amounts of the particles into the tissues. The rapid nanoparticle uptake observed demonstrates that these lipid particles are promising vehicles to accomplish both local and systemic drug delivery following nasal administration.

## 1. Introduction

Lipid-associated delivery systems for nasal administration, including liposomes, solid lipid nanoparticles (SLN), and nanostructured lipid carriers (NLC), have shown promise in the delivery of vaccines, systemically available therapeutics, and as targeting strategies to reach the CNS via the nose-to-brain pathways [1]. These lipid-associated systems can protect their drug or vaccine payloads from enzymatic degradation from the variety of metabolically active enzyme systems in the nasal secretions and mucosa, including cytochrome P450s, monooxygenases, carboxylesterases and a number of conjugative enzyme systems [2,3,4]. Nanoparticles can also enable escape from undesirable, transporter-mediated efflux; they can mask unsuitable drug properties; and can provide additional pathways for entering the nasal mucosa. Lipid-based nanoparticles suitable for drug delivery are typically 50–200 nm in size, and a variety of surface-associated ligands have been investigated to improve their cellular uptake and targeting [1,5,6]. While a number of strategies have shown preliminary success in delivering lipid-based nanoparticles to the nasal mucosa or via the nasal mucosa on to the brain, little is known about the specific mechanisms of lipid-associated particle uptake [7]. Nanoparticles, including lipid-based nanoparticles, typically enter cells via the processes collectively known as “endocytosis”. However, smaller (≤50 nm) nanoparticles have also been reported to pass through the paracellular pathway [8,9]. There are a number of specific endocytic processes, including clathrin-mediated endocytosis (CLME), caveolae-mediated endocytosis (CVME) and macropinocytosis (MP), and many of the endocytic processes are further associated with the endosomal/lysosomal pathways of cellular trafficking. As a result, depending on their lipid compositions and other surface characteristics, lipid-based nanoparticles can undergo uptake followed by later release from endosomes, the nanoparticles may undergo lysosomal degradation, or they may be released from the cell via extracellular vesicles/exosomes [10]. Depending on the delivery target for the nanoparticle drug payload, intracellular release in the nasal epithelium may achieve the desired therapeutic outcome, but in many situations, further distribution of the drug-containing nanoparticles to more distant tissue sites is desired.

Very little is currently known about the activities of the various endocytic processes driving the uptake of nanoparticles in the nasal mucosa, and the current investigations were undertaken to probe the activity of CLME, CVME, and MP in the uptake of solid lipid nanoparticles carrying a lipophilic fluorescent dye, Nile Red, which enabled the quantification of nanoparticle uptake in nasal tissues. For these initial investigations, the SLNs did not contain any specific targeting moieties, but a broad range of SLN sizes, from 30 to 150 nm, were investigated to determine whether there were size-dependent pathways available for targeted SLN uptake in the nasal mucosa.

## 2. Materials and Methods

### 2.1. Materials

Stearic acid, heneicosane, Nile Red, poloxamer 188 (Kolliphor^®^ P-188), polyoxyethylene (10) oleyl ether (Brij^®^ O10), nonaethylene glycol monododecyl ether (L-9), polysorbate 80 (Tween^®^ 80), butyric acid, 2-ethoxyethyl acetate (Cellosolve^®^ acetate), trypsin-EDTA solution containing 2.5 g of porcine trypsin and 0.2 g of EDTA in 500 mL Hank’s balanced salt solution, chlorpromazine hydrochloride, amiloride hydrochloride, 2,4-dinitrophenol, filipin, and 2-ethoxyethyl acetate (Cellosolve^®^ acetate) were obtained from Sigma-Aldrich, Co. (St. Louis, MO, USA). Soy lecithin was obtained from Alfa Aesar Co. (Sparks, NV, USA). Dichloromethane, dimethyl sulfoxide (DMSO) and sodium azide were obtained from Fisher Chemicals (Fair Lawn, NY, USA). Methyl-β-cyclodextrin (MBCD) was purchased from Acros Organic (Morris Plains, NJ, USA). Krebs Ringer bicarbonate buffer (KRB) was prepared using 123.8 mM sodium chloride, 1.5 mM monobasic sodium phosphate, 4.56 mM potassium chloride, 10 mM dextrose, and 15 mM sodium bicarbonate, all obtained from Research Products International Corp (Mt. Prospect, IL, USA), and 1.67 mM magnesium chloride, 0.7 mM dibasic sodium phosphate, and 1.2 mM calcium chloride, which were obtained from Sigma Aldrich Co. (St. Louis, MO, USA). The buffer pH was adjusted to 7.4 ± 0.05 with 1 N sodium hydroxide solution (VWR, Radnor, PA, USA).

### 2.2. Preparation of 150 nm Solid Lipid Nanoparticles Using Emulsion/Solvent Evaporation

Solid lipid nanoparticles (150 nm) were prepared using an emulsion/solvent evaporation method modified from Naguib et al. [11]. The organic phase was prepared by dissolving 40 mg stearic acid and 30 mg soy lecithin in 1 mL dichloromethane containing 100 μg Nile Red. This organic phase was added directly to the aqueous phase (10 mL containing 1% *w*/*v* poloxamer 188 and 50 μL butyric acid), and an ultrasonic probe sonicator (Model 100, Fisher Scientific, Pittsburgh, PA, USA) operated at 75% amplitude for 6 min was used to form the SLNs. The dispersion was kept in an ice bath during SLN formation and then moved to a water bath (48 ± 2 °C) for 15 min with stirring at ~300 rpm. Finally, the dispersion was placed in a fume hood at room temperature with stirring at ~200 rpm for one hour to ensure complete dichloromethane evaporation. The dispersion was filtered through Whatman^®^ filter paper (# 541, Global Life Sciences Solutions, Pittsburgh, PA, USA) under vacuum to remove any large, non-emulsified solids. This was followed by filtration through a 0.45 μm syringe filter (mixed cellulose esters membrane (SLHA033SS), Merck Millipore Ltd., Carrigtwohill, Ireland). The filtered dispersion was combined with 5 mL Nanopure^®^ water and placed in an Amicon^®^ Ultra-15 centrifugal filter unit (Merck Millipore Ltd., Ireland) and centrifuged (Eppendorf (Model 5810R), Hauppauge, Suffolk County, NY, USA) at 500× *g* for 45 min at 4 °C to separate free dye and excess surfactant from the particles. The solid lipid nanoparticle suspension was filtered through a 0.22 μm syringe filter (MCE, Merck Millipore Ltd., Ireland) to remove any aggregated and/or larger particles.

### 2.3. Preparation of 60 and 30 nm Solid Lipid Nanoparticles Using Phase Inversion

Solid lipid nanoparticles (30 and 60 nm) were prepared using a phase inversion temperature method as previously described [12]. To prepare the 60 nm SLNs, heneicosane (100 mg) was combined with Nile Red (600 μg) and co-melted at 90 ± 2 °C in a water bath with stirring. Nonaethylene glycol monododecyl ether (L-9) (200 μL) was added along with 1.79 mL Nanopure^®^ water. The mixture was kept at 90 ± 2 °C until a “semi” phase separation occurred. The mixture was removed from the water bath and vortexed followed by shaking for 15 min at room temperature. The resulting nanoemulsion was cooled at 4 °C for 15 min to aid in the solidification of the lipid phase of the nanoparticles. The nanoparticle dispersion was then filtered through a 0.22 μm syringe filter (MCE, Merck Millipore Ltd., Ireland), combined with Nanopure^®^ water (7 mL) and the mixture was placed in an Amicon^®^ Ultra-15 centrifugal filter unit (Merck Millipore Ltd., Ireland). The unit was centrifuged (Eppendorf model 5810R, Hauppauge, NY, USA) at 500× *g* for 45 min at 4 °C to concentrate the nanoparticle dispersion within the filter unit. The solid lipid nanoparticle dispersion was filtered through a 0.22 μm syringe filter (MCE membrane (GSWP04700), Merck Millipore Ltd., Ireland) to remove any remaining aggregated and/or large particles, and the filtrate was collected for further use. For the 30 nm SLNs, the L-9 surfactant was replaced with polyoxyethylene (10) oleyl ether (Brij^®^ O10) (150 μL). The remaining preparation steps were the same as for the 60 nm SLNs.

### 2.4. Measurement of Size, Shape, and Zeta Potential

The size and surface charge (zeta potential) of the SLNs were measured using a Malvern Nano-ZS Zetasizer (Worcestershire, UK). Samples were analyzed for particle size using a disposable cuvette (DTS0012, Malvern Instruments Inc., Westborough, MA, USA). The zeta potential measurement was conducted using a folded capillary cell (DTS1070, Malvern Instruments Inc., Westborough, MA, USA). Measurements were made following dilution in Nanopure^®^ water, KRB, or KRB + inhibitor solutions. The solid lipid nanoparticles were negatively stained with 2% phosphotungstic acid and particle morphology was examined using a JEOL EM–1230 transmission electron microscope (JEOL, Peabody, MA, USA).

### 2.5. Loading and Encapsulation Efficiency

Batches of the SLN dispersions were freeze-dried (VirTis ADVANTAGE, SP Industries, Warminster, PA, USA) to measure the yield and the Nile Red loading in the SLNs (freshly prepared SLNs were used for the transport experiments). Lyophilized SLNs were dissolved in 2-ethoxyethyl acetate to give a concentration of 1 mg/mL of the nanoparticles, and the mixture was incubated for 48 h in the dark at 37 °C with shaking. Similarly, 1 mg of Nile Red was dissolved in 20 mL of 2-ethoxyethyl acetate to prepare a control solution. The content of Nile Red in the SLNs was determined using a SpectraMax M5 Multi-Mode Microplate Reader (Molecular Devices, Sunnyvale, CA, USA). The fluorescence intensities of Nile Red in the standard and samples were measured at an excitation wavelength of 530 nm and an emission wavelength of 600 nm. The SLN loading was calculated using Equation (1) and the encapsulation efficiency was calculated using Equation (2).

(1) $$\begin{array}{l} \mathrm{Loading}\;\left(\frac{\mu g\;\mathrm{of}\;\mathrm{dye}}{\mathrm{mg}\;\mathrm{of}\;\mathrm{nanoparticles}}\right)\\ \;\;\;\;\;\;\;\;=\frac{\mathrm{calculated}\;\mathrm{dye}\;\mathrm{concentration}\;\left(\mu g/\mathrm{mL}\right)*\mathrm{sample}\;\mathrm{volume}\;\left(\mathrm{mL}\right)}{\mathrm{weight}\;\mathrm{of}\;\mathrm{nanoparticles}\;\mathrm{used}\;\left(\mathrm{mg}\right)} \end{array}$$

(2) $$\mathrm{Encapsulation}\;\mathrm{Efficiency}\;\left(\%\right)=\frac{\mathrm{mass}\;\mathrm{of}\;\mathrm{dye}\;\mathrm{in}\;\mathrm{nanoparticles}\;\left(\mu g\right)}{\mathrm{intial}\;\mathrm{mass}\;\mathrm{of}\;\mathrm{dye}\;\mathrm{in}\;\mathrm{the}\;\mathrm{system}\;\left(\mu g\right)}*100\%$$

The nanoparticle loading was used to convert the amount of Nile Red recovered from the nasal tissues to an equivalent mass of SLNs containing that amount of dye. This allowed for the comparison of the amounts of nanoparticles transferred into the nasal tissues rather than simply comparisons of the concentrations of the marker dye compound. Preliminary investigations were conducted to evaluate the release of Nile Red from the SLNs, and due to the hydrophobic natures of both the dye and the SLNs, <5% of the dye was released during a 30 min period, similar to the length of time of the incubation period in the uptake studies, indicating that all of the Nile Red recovered from the nasal tissues was associated with the SLNs present in the tissues.

### 2.6. Bovine Nasal Olfactory and Respiratory Tissues

Fresh bovine olfactory and respiratory nasal tissues were obtained from a local abattoir (Bud’s Custom Meats Co., Riverside, IA, USA). Tissues were excised from the nasal cavities of decapitated cows by making incisions along the lateral walls of the nasal cavity to harvest the respiratory tissues and a vertical incision was made perpendicular to the ocular plane to harvest the olfactory tissues. After harvesting, the tissues were placed in ice-cold KRB and transported on ice to the lab.

### 2.7. Nanoparticle Uptake Studies

Small tissue sections (epithelium + submucosa) were carefully peeled away from the underlying cartilage and mounted between the donor and receiver chambers of NaviCyte^®^ diffusion chambers (Warner Instruments, LLC, Hamden, CT, USA) with the epithelial layer facing the donor side. After mounting the tissues, 1 mL pre-warmed (37 °C) KRB was placed in the donor and receiver chambers and the tissues were equilibrated for 1 h at 37 °C. Stirring with carbogen (O_2_/CO_2_, 95%/5%) at a rate of 1–2 bubbles per second was used during the equilibration time and throughout the experiments. After equilibration, the donor chamber was replaced with 1 mL of 0.1% *w*/*v* SLNs (30, 60, or 150 nm) in KRB and the receiver chamber was replaced with 1 mL pre-warmed KRB; nanoparticle uptake proceeded during the following 30 min. The integrity of the exposed tissues was evaluated by measuring the transepithelial electrical resistance (TEER) at the beginning of the equilibration time and near the end of the transport experiments using an EVOM2 epithelial volt-ohmmeter (World Precision Instruments, Sarasota, FL, USA). At the end of each experiment, the tissues were removed from the diffusion chambers, rinsed with deionized water, and the regions of the tissues exposed to the nanoparticle dispersion were removed for further processing. Each exposed tissue section was placed into a 15 mL polypropylene tube containing 2 mL of trypsin-EDTA solution and incubated for 2 h at room temperature with shaking to remove the epithelial cells from the underlying submucosal tissues.

At the end of the incubation time, the remaining submucosal tissue section was removed from the epithelial cell dispersion and transferred to a separate, empty 15 mL polypropylene tube. The amounts of nanoparticles present in the nasal tissues were determined by digesting the epithelial cell dispersions and submucosal tissues with 1.5 mL of 2-ethoxyethyl acetate in each tube and incubating the tubes at 37 °C in the dark with shaking for 48 h. The samples were brought to room temperature and centrifuged for 5 min at 4 °C at 2000× *g* (Eppendorf Model 5810R, Hauppauge, NY, USA). The Nile Red was released from the dissolved nanoparticles and was present in the 2-ethoxyacetate layer at the top of the contents in the tube. The samples were allowed to return to room temperature and the fluorescence intensity of the released dye in the 2-ethoxyethyl acetate phase was measured at Ex: 530/Em: 600 nm.

### 2.8. SLN Uptake Determination Using Pharmacological Inhibitors of Endocytosis

#### 2.8.1. 150 nm Solid Lipid Nanoparticles

To identify the involvement of each of the endocytic pathways in the cellular entry of the 150 nm SLNs, nasal tissues mounted in the NaviCyte^®^ diffusion chambers were pre-treated with chemical inhibitors of endocytosis during the pre-incubation period (1 h). Chlorpromazine (CPZ, 10 μg/mL) was used to inhibit clathrin-mediated endocytosis (CLME) [13]; amiloride (266 μg/mL) was used to inhibit macropinocytosis (MP) [14]; methyl-β-cyclodextrin (MBCD, 5 mg/mL) was used to inhibit caveolae-mediated endocytosis (CVME) [13]; and 2,4-dinitrophenol (2,4-DNP) (184 μg/mL, 1.84 mg/mL) or sodium azide (150 μg/mL, 650 μg/mL) were used to inhibit ATP synthesis [15]. The inhibitor-containing solutions in the donor chamber were removed and replaced with SLN dispersions and the receiver chamber solutions were replaced with fresh KRB. In the case of 2.4-DNP and sodium azide, the metabolic inhibitors were also included with the SLN dispersions during the incubation period. After 30 min of incubation with the 150 nm SLNs, the tissues were removed from the diffusion cells, rinsed with deionized water, and the exposed areas were recovered and processed as described in the Nanoparticle Uptake Studies section.

#### 2.8.2. 60 nm Solid Lipid Nanoparticles

Uptake of the 60 nm particles was evaluated using the same methods as for the 150 nm SLNs except the inhibitors were also included with the SLN dispersions during the incubation period, in addition to their inclusion in the pre-incubation medium. For the 60 nm SLNs, CVME was evaluated using filipin (5 μg/mL) instead of methyl-β-cyclodextrin. Dimethyl sulfoxide (DMSO) (1%) was used for adequate filipin solubility, and 1% DMSO was also included in the pre-incubation medium. Filipin was not included with the SLN dispersions during the uptake exposure period due to the ability of DMSO to solubilize the SLNs and Nile Red.

#### 2.8.3. 30 nm Solid Lipid Nanoparticles

For the investigation of the endocytic uptake of 30 nm SLNs, the nasal tissues were pre-treated with the inhibitors in the pre-incubation medium without their inclusion with the SLN dispersions (“pre-treated”) or the inhibitors were included both in the pre-incubation medium and with the SLN dispersions (“included”). For 2,4-DNP, a concentration of 184 μg/mL was used for tissue pre-incubation and with the SLNs, or a higher concentration (1.84 mg/mL) was used with the SLNs in a follow-up set of experiments. Sodium azide (150 or 650 µg/mL) was used for pre-incubation and was included at the same concentrations in the medium containing the 30 nm SLNs. Filipin in 1% DMSO was used to investigate CVME by pre-treating the tissues with the inhibitor solution in a similar manner as for the 60 nm SLNs.

## 3. Results

**Figure 1:**
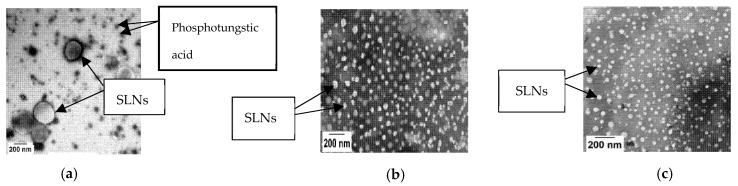
Transmission electron micrographs of 150 nm (**a**), 60 nm (**b**), and 30 nm (**c**) SLNs. The nanoparticles were negatively stained with 2% phosphotungstic acid. A JEOL JEM-1230 TEM (JEOL, Peabody, MA, USA) was used to obtain the images.

The Nile Red-containing SLNs were observed to be spherical in shape (Figure 1), each size showed reasonable polydispersity (Table 1), and the zeta potentials measured in KRB were slightly negative (Table 1).

**Figure 2:**
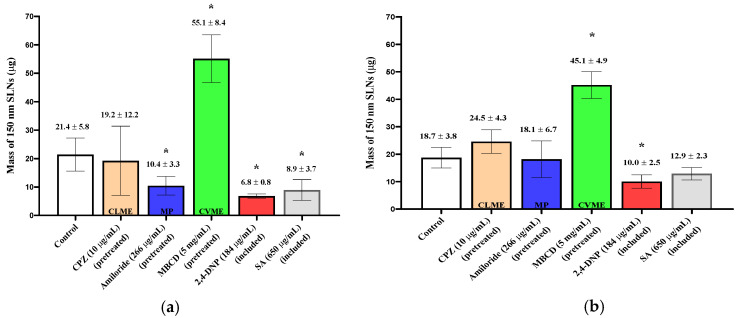
Uptake (30 min) of 150 nm SLNs in the nasal olfactory (**a**) and respiratory (**b**) tissues pre-treated with endocytic inhibitors. 2,4-DNP and sodium azide were also included with the SLNs. Bar graphs display mean nanoparticle uptake ± standard deviation determined from the recovery of Nile Red from the tissues. Statistical comparisons were made between each of the inhibited tissues and the control tissues using Student’s two-tailed *t*-test (*p* < 0.05). * denotes a statistical difference between the inhibited and control tissues.

**Figure 3:**
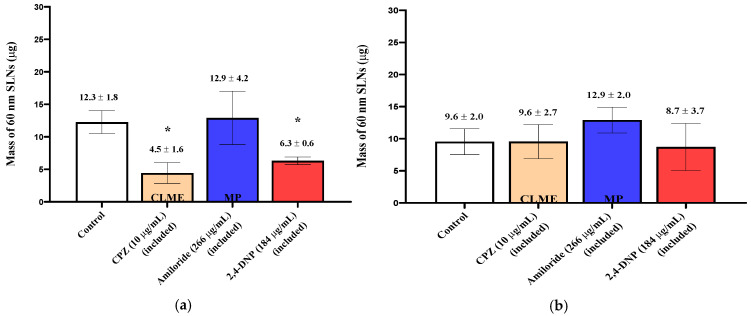
Uptake (30 min) of 60 nm SLNs in the nasal olfactory (**a**) and respiratory (**b**) tissues in the presence of endocytic inhibitors. Bar graphs display mean nanoparticle uptake ± standard deviation determined from the recovery of Nile Red from the tissues. Statistical comparisons were made between each of the inhibited tissues and the control tissues using Student’s two tailed *t*-test (*p* < 0.05). * denotes a significant statistical difference between the inhibited and control tissues.

**Figure 4:**
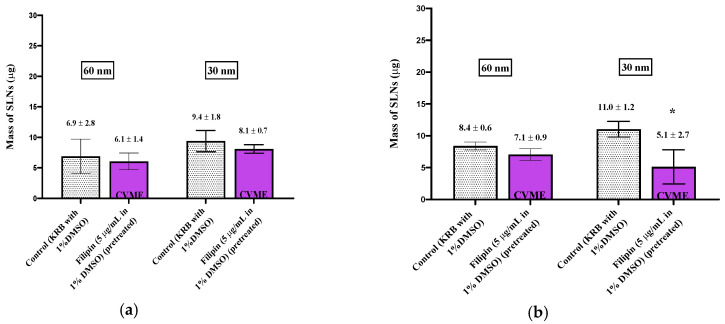
Uptake (30 min) of 60 and 30 nm SLNs in the nasal olfactory (**a**) and respiratory (**b**) tissues pre-treated with filipin. Bar graphs display mean nanoparticle uptake ± standard deviation determined from the recovery of Nile Red from the tissues. Statistical comparisons were made between the inhibited and control tissues using Student’s two-tailed *t*-test (*p* < 0.05). * denotes a significant statistical difference between the inhibited and control tissues.

When the 150 nm SLNs (in KRB) were incubated with the excised nasal tissues, ~20 µg of the particles were taken up into the tissues (Figure 2), and for the 30 and 60 nm SLNs, slightly more than 10 µg of the nanoparticles were transferred into the tissues (Figure 3; Figure 4). For both tissue types and all nanoparticle sizes, the amount of SLNs taken up by the tissues was less than 2.5% of the amount of SLNs present in the incubation medium. Comparing the uptake between the control respiratory and olfactory tissues, the uptake of the SLNs in the olfactory tissues was comparable or slightly higher compared to that in the respiratory tissues. While the nanoparticle concentrations in the epithelial cell layer and the submucosal tissues were measured separately, for clarity, the total amounts of nanoparticles in the entire tissue (sum of epithelial and submucosal amounts) are reported and compared following exposure to the pharmacologic inhibitors of endocytosis.

When the olfactory tissues were exposed to chlorpromazine (CPZ; 10 μg/mL), a clathrin-mediated endocytosis (CLME) inhibitor during the pre-incubation period, the uptake of the 150 nm SLNs was unaffected, but their uptake was significantly reduced (~50%) by amiloride, a macropinocytosis (MP) inhibitor (Figure 2). In the nasal respiratory tissues, neither chlorpromazine nor amiloride reduced the uptake of the 150 nm SLNs. A surprising increase in nanoparticle uptake in both tissue types was observed following pre-treatment with methyl-β-cyclodextrin (MBCD), and this increase was consistently observed with all of the SLNs investigated (results not shown). Since MBCD is also able to solubilize membrane components and affect tight junction integrity, it appears that these direct effects on the membrane overshadowed any potential inhibition of caveolae-mediated endocytosis. To further investigate endocytic activity in the nasal mucosa, two general metabolic inhibitors used to inhibit all energy-dependent cellular processes were also investigated by both pre-treating the tissues and continuing to expose them to the inhibitors during SLN incubation (“included”). Exposure to 2,4-DNP reduced nanoparticle uptake in the olfactory tissues by ~68%, while sodium azide lowered it by ~59%. In the respiratory tissues, 2,4-DNP reduced the uptake by ~42%, while sodium azide lowered it by ~31%. These results confirm that a significant proportion of SLN uptake is initiated by energy-dependent mechanisms, as is expected for endocytic uptake [16].

When smaller (60 nm) nanoparticles were investigated, new patterns of uptake and inhibition were observed. During the experiments with 60 nm SLNs, the inhibitors (except filipin) were included in both the pre-incubation media and with the SLNs in the incubation media in order to prolong cellular exposure to the inhibitors. After a 30 min incubation with 60 nm SLNs in the presence of CPZ, a reduction (64%) in SLN uptake by the olfactory tissues was observed, but no change in uptake occurred in the respiratory tissues (Figure 3). No differences, compared to control, were observed in the amiloride-treated olfactory and respiratory tissues. Filipin was used to evaluate CVME, and no change in the uptake of the 60 nm SLNs in the nasal olfactory or respiratory tissues was observed using this inhibitor. Additional control tissues exposed to the 1% DMSO-KRB used as the pre-incubation medium were included for comparison with the filipin-inhibited tissues; these experimental results are displayed separately from the other pharmacologic inhibitors (Figure 4). The ATP synthesis inhibitor, 2,4-DNP, reduced the uptake of the 60 nm SLNs by ~50% in the olfactory tissues, but not in the respiratory tissues (Figure 3). This was similar to the results for the 150 nm SLNs, where the uptake by the olfactory tissues was affected to a greater extent than the respiratory tissues.

**Figure 5:**
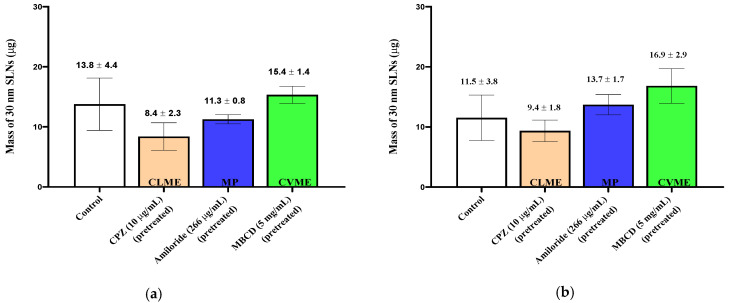
Uptake (30 min) of 30 nm SLNs in the nasal olfactory (**a**) and respiratory (**b**) tissues pre-treated with endocytic inhibitors. Bar graphs display mean nanoparticle uptake ± standard deviation determined from the recovery of Nile Red from the tissues. Statistical comparisons were made between each of the inhibited tissues and control tissues using Student’s two-tailed *t*-test (*p* < 0.05). No statistically significant differences were found.

**Figure 6:**
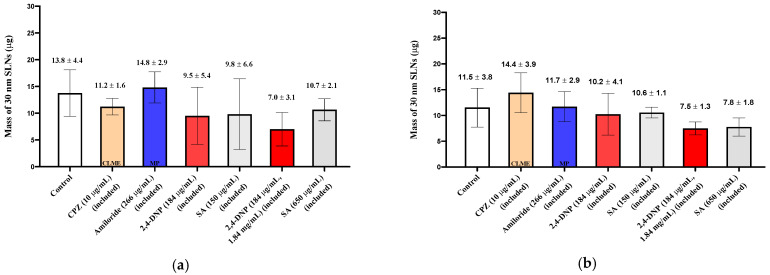
Uptake (30 min) of 30 nm SLNs in the nasal olfactory (**a**) and respiratory (**b**) tissues in the presence of endocytic inhibitors during the 30 min nanoparticle incubation period. Bar graphs display mean nanoparticle uptake ± standard deviation determined from the recovery of Nile Red from the tissues. Statistical comparisons were made between each of the inhibited tissues and the control tissues using Student’s two-tailed *t*-test (*p* < 0.05). No statistically significant differences were found.

The uptake and quantification of 30 nm SLNs in the excised nasal tissues was investigated by exposing the tissues to the inhibitors in the pre-incubation medium (“pre-treated”) or including the inhibitor in the pre-incubation media and in the SLN dispersion. In both cases, no reductions in the 30 nm SLN uptake were observed for any of the inhibitors in either nasal tissue type (Figure 5; Figure 6), except in the case of filipin pre-treatment, where the nasal respiratory tissues showed a reduction in uptake of ~50% (Figure 4).

In addition to measuring the uptake of SLNs into the nasal mucosal tissues, the receiver chambers of the NaviCyte^®^ cells were sampled to measure SLN transfer through and exit from the tissues. The Nile Red fluorescence measurements in the receiver chamber were indistinguishable from the control KRB medium, indicating that virtually no nanoparticles exited the excised tissues. Since there are limited mechanisms or pathways available in the submucosal tissues to assist in the transfer of the nanoparticles out of the tissues, the lack of appearance of materials in the receiver chamber should not be interpreted to mean that there will be no systemic transfer of those particles in vivo. Since the blood and lymph vessels involved in the systemic distribution of materials absorbed via the nasal mucosa lie within the submucosal region, transfer into these vessels is likely, even if escape from the tissues in the experimental system was not observed.

## 4. Discussion

While the SLN uptake results show no consistent pattern of involvement of individual endocytic uptake mechanisms, the results clearly indicate that energy-dependent uptake processes are involved in SLN uptake. Classic pharmacologic inhibitors of clathrin or caveolae-mediated endocytosis and of macropinocytosis were able to reduce the uptake of nanoparticles under specific circumstances (60 nm SLNs in olfactory tissues; 30 nm SLNs in respiratory tissues; and 150 nm SLN’s in olfactory tissues, respectively), but none of these inhibitors was able to completely eliminate the uptake of the SLNs, even if they were able to affect a moderate decrease in total transfer. Less specific metabolic inhibitors (2,4-DNP and sodium azide) were used to probe a broader spectrum of energy-dependent mechanisms in the tissues, some of which play a role in nanoparticle uptake. Even the use of these more general inhibitors did not accomplish the complete abolishment of nanoparticle uptake, although they were able to significantly reduce the uptake of the larger, 150 and 60 nm, SLNs.

Previous investigators have also reported on pharmacologic inhibitors reducing, but not completely eliminating, the uptake of SLNs of similar sizes in a variety of other epithelial cell types. When investigating the uptake of relatively large (~150 nm) SLNs, Patel et al. used CPZ (10 µg/mL) to probe the role of clathrin-mediated endocytosis on the uptake of 140 nm SLNs in Caco-2 cells. They reported the uptake of the SLNs to be significantly reduced (60%) but not completely inhibited in the CPZ-treated cells [17]. Neves et al., however, reported that the uptake of 180 nm SLNs in pre-treated Caco-2 cells was not reduced by cytochalasin-D (5 μg/mL), an inhibitor of macropinocytosis with moderate inhibitory activity for CLME and CVME [18]. Similarly, Wonganan et al. reported that the uptake of 146 nm SLNs by the mouse lung cancer (TC-1-GR) cell line was not reduced upon employing cytochalasin-D (20 ng/mL) [19]. When investigating the role of CVME in epithelial cells, Wonganan et al. also reported that the uptake of 146 nm SLNs by TC-1-GR cells was not reduced following exposure to filipin (2.5 μg/mL) [19], but Patel et al. observed that nystatin (50 μg/mL) significantly reduced (45%) the uptake of 140 nm SLNs across Caco-2 cells. Patel et al. also investigated 140 nm SLN uptake using the metabolic inhibitor, sodium azide (1 mg/mL), and observed an 85% reduction in SLN uptake [17]. Similarly, Shah et al. also reported a 37% reduction in the uptake of 160 nm SLNs by Caco-2 cells using sodium azide (1 mg/mL) [20]. All of these results continue to suggest that inhibiting a single endocytic pathway has a limited effect on the overall endocytic activity of the epithelial cells.

Similar to the results observed for 150 nm SLNs, variable effects of pharmacologic inhibitors on the uptake of ~60 nm SLNs in a variety of epithelial cells have also been reported. CLME is a very active endocytic process that has been reported to be highly involved in the uptake of a wide range of nanoparticles with diameters typically up to 200 nm [21], and reports of the contribution of this route in the uptake of the SLNs in the ~50–90 nm size range have been described using a variety cell lines including Caco-2 cell monolayers [43% inhibition in the presence of CPZ (10 μg/mL)] [22]. In a separate study, Chai et al. also reported that the uptake of the same SLNs was significantly inhibited (30%) by CPZ (10 μg/mL) in MDCK cell monolayers [15]. Similarly, Li et al. also reported that the uptake of 70 nm SLNs in MDCK-II cells was significantly reduced (35%) when employing CPZ (10 μg/mL) [23]. When examining nanoparticle uptake in excised tissues instead of using cell cultures, Zhang et al. showed the uptake of 50 and 70 nm SLNs was significantly inhibited (40%) by CPZ (10 μg/mL) in an everted rat intestinal ring system [24]. In the current studies using excised nasal tissues, inhibition of CLME by CPZ was only observed in the olfactory tissues for the 60 nm SLNs. However, this lack of inhibition does not suggest that CLME is not occurring in the excised nasal tissues, but instead suggests that the combination of all endocytic processes and other available uptake pathways are sufficient to maintain a significant uptake of SLNs despite the transient inhibition of one of the endocytic pathways.

Macropinocytosis can also play a role in the uptake of moderately sized (50–90 nm) SLNs, but based on previous investigations, the type of cell monolayer appears to influence the extent of utilization of this endocytic pathway. For example, Chai et al. studied the same (87 nm) SLNs as investigated with CPZ but, instead used 5-(*N*-ethyl-*N*-isopropyl) amiloride (EIPA, a MP inhibitor; 30 μg/mL) and found that SLN uptake was not reduced in an MDCK cell monolayer, but it was reduced (29%) in a Caco-2 cell monolayer [15,22]. These results suggest, however, that even when MP plays a role in the uptake of SLNs in selected cell-types, its role is likely in combination with multiple other endocytic uptake mechanisms. Amiloride exposure showed no effect on the uptake of 60 nm SLNs in either olfactory or respiratory tissues in the current investigations, further supporting the previous observations of the limited reductions in SLN uptake in the presence of amiloride.

Like CLME and MP, other reports about the inhibition of the uptake of moderately sized nano-carriers by CVME vary regarding the extent of inhibition. Li et al. reported that the uptake of 70 nm SLNs in MDCK-II cells was only reduced by 15% when employing filipin (~1 µg/mL) [23], but Chai et al. reported that the uptake of 87 nm SLNs by an MDCK cell monolayer was significantly inhibited (50%) by filipin (5 µg/mL) and nystatin (28 µg/mL) (30%) [15]. Similar results were also reported using excised tissues by Zhang et al. who showed the uptake of 50 and 70 nm SLNs was significantly reduced (30%) by nystatin (25 µg/mL) (a CVME inhibitor) in everted rat intestinal rings [24]. In comparison, in the excised nasal tissues examined in these studies, no decrease in 60 nm SLN uptake was observed when the tissues were pre-treated with filipin.

Reports of the effects of pharmacologic inhibitors on the uptake of very small (<50 nm) SLNs in epithelial cells are limited, but unlike in the current investigations where no significant reduction in the uptake of 30 nm SLNs was observed with any inhibitor, Zhang et al. reported that the uptake of 24 nm SLNs significantly inhibited (38%) from entering Caco-2 cells, and 48 nm SLN uptake was also significantly reduced (44%) in an everted rat intestinal ring system by CPZ (10 µg/mL) [24,25]. In addition, inhibition of CVME with nystatin (25 µg/mL) also resulted in a reduced (30%) uptake of 48 nm SLNs in the everted rat intestinal rings [25].

The differences reported among investigations of the endocytic uptake mechanisms involved in SLN uptake by epithelial tissues may be the result of the variety of different experimental methods used by different investigators, including whether there was ongoing exposure to the inhibitor in the SLN media, the inhibitor concentration, the time allowed for uptake, and the differences among the wide variety of cell types and tissues studied. Of significant importance to the drug delivery applications of these results, however, is the observation that none of the reported studies accomplished the complete abolishment of the uptake of SLNs through the use of a single pharmacologic inhibitor. Due to the complexity of the mechanisms involved in the cellular uptake of endogenous and exogeneous substances, it is not surprising that a number of pathways appear to be available to most nanoparticles, including the SLNs used in these investigations. It is likely that most particles will have access to many of the endocytic pathways, enabling them to enter epithelial cells, including those present on the surfaces of the olfactory and respiratory nasal mucosae, assuring the uptake of at least limited amounts of the nanoparticles and their drug payloads. Additional non-energy-dependent pathways also appear to be available for the uptake and transfer of nanoparticles in nasal tissues as demonstrated by the continued transfer of nanoparticles into the tissues in the presence of potent metabolic inhibitors. The nasal mucosa is a well-recognized “leaky” mucosa with limited intercellular barriers, as exemplified by the low TEER values measured across the nasal epithelium, both in primary cell cultures and in the excised tissues used in these experiments [26]. These intercellular pathways may offer additional opportunities for particle transfer into the tissues, especially for the smallest (30 nm) SLNs, which showed high levels of nanoparticle uptake that were unaffected by the presence of any of the endocytic inhibitors tested.

## Figures and Tables

**Table 1:** Solid lipid nanoparticles size distribution and zeta potential measurements. Data are presented as mean ± standard deviation.

Target Mean Particle Diameter	Mean (z-Average) Particle Diameter (nm)		Polydispersity Index (PDI)		Zeta Potential (mV)		Yield (%)	Entrapment Efficiency (%)
	Nanopure Water	KRB	Nanopure Water	KRB	Water	KRB		
150 nm	145.3 ± 8.6	174.0 ± 1.2	0.14 ± 0.02	0.19 ± 0.01	−34.6 ± 2.5	−15.4 ± 1.0	19	25
60 nm	66.4 ± 1.4	62.7 ± 7.2	0.27 ± 0.05	0.33 ± 0.07	−5.8 ± 0.2	−3.8 ± 0.1	73	73
30 nm	32.3 ± 3.6	34.6 ± 4.7	0.26 ± 0.08	0.16 ± 0.08	−11.7 ± 1.7	−2.7 ± 0.4	69	60
